# Glucose-Dependent Promoters for Dynamic Regulation of Metabolic Pathways

**DOI:** 10.3389/fbioe.2018.00063

**Published:** 2018-05-22

**Authors:** Jérôme Maury, Soumya Kannan, Niels B. Jensen, Fredrik K. Öberg, Kanchana R. Kildegaard, Jochen Forster, Jens Nielsen, Christopher T. Workman, Irina Borodina

**Affiliations:** ^1^Novo Nordisk Foundation Center for Biosustainability, Technical University of Denmark, Lyngby, Denmark; ^2^Department of Biotechnology and Biomedicine, Technical University of Denmark, Lyngby, Denmark

**Keywords:** inducible promoters, dynamic regulation, yeast *Saccharomyces cerevisiae*, strain engineering, 3-hydroxypropionic acid

## Abstract

For an industrial fermentation process, it can be advantageous to decouple cell growth from product formation. This decoupling would allow for the rapid accumulation of biomass without inhibition from product formation, after which the fermentation can be switched to a mode where cells would grow minimally and primarily act as catalysts to convert substrate into desired product. The switch in fermentation mode should preferably be accomplished without the addition of expensive inducers. A common cell factory *Saccharomyces cerevisiae* is a Crabtree-positive yeast and is typically fermented at industrial scale under glucose-limited conditions to avoid the formation of ethanol. In this work, we aimed to identify and characterize promoters that depend on glucose concentration for use as dynamic control elements. Through analysis of mRNA data of *S. cerevisiae* grown in chemostats under glucose excess or limitation, we identified 34 candidate promoters that strongly responded to glucose presence or absence. These promoters were characterized in small-scale batch and fed-batch cultivations using a quickly maturing rapidly degrading green fluorescent protein yEGFP3-Cln2_PEST_ as a reporter. Expressing 3-hydroxypropionic acid (3HP) pathway from a set of selected regulated promoters allowed for suppression of 3HP production during glucose-excess phase of a batch cultivation with subsequent activation in glucose-limiting conditions. Regulating the 3HP pathway by the *ICL1* promoter resulted in 70% improvement of 3HP titer in comparison to *PGK1* promoter.

## Introduction

**Figure 1:**
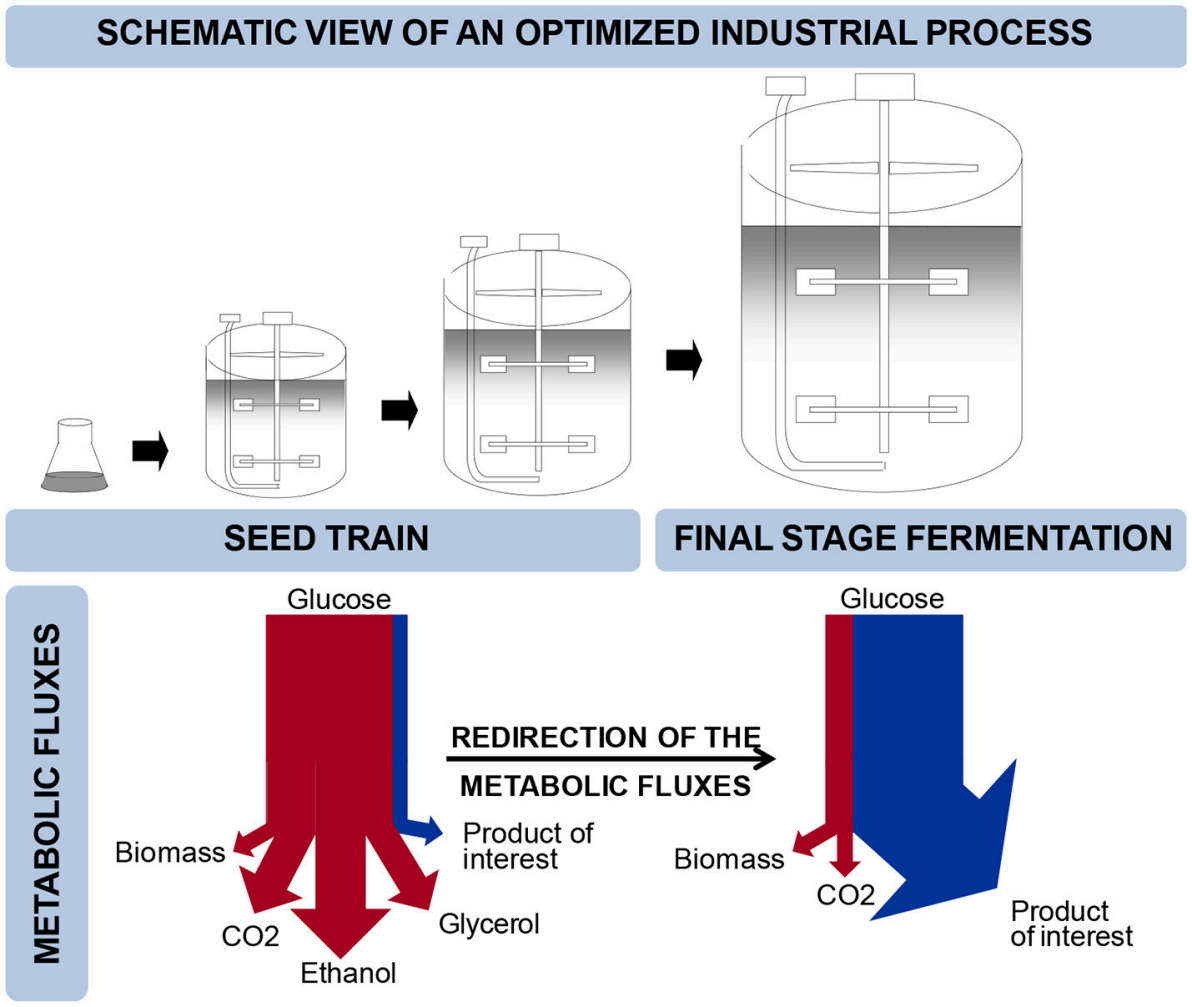
Schematic view of an optimized industrial process. The top panel illustrates the progression of fermentation settings from the seed train to full scale production. The lower panel illustrates the corresponding metabolic fluxes and the switch from biomass generation to product formation.

Budding yeast, *Saccharomyces cerevisiae*, is widely used for the production of fuels, chemicals, food ingredients, food and beverages, and pharmaceuticals (Nielsen and Jewett, 2008; Hong and Nielsen, 2012). *S. cerevisiae* is further being exploited for production of many new products, e.g., farnesene, butanol, resveratrol or melatonin (Hong and Nielsen, 2012; Shin et al., 2012; Germann et al., 2016; Meadows et al., 2016) and many more are under development. The enabling technology in the development of yeast cell factories is metabolic engineering, which is the introduction of directed genetic modifications with the objective to improve the performance of the cell factory (Nielsen and Jewett, 2008). Development of cell factories that can meet the high requirements for yield, titer and productivity, generally requires multiple rounds of metabolic engineering. In connection with implementation of cell factories for industrial production, it is necessary to scale-up the process, and the final process will typically involve subsequent rounds of cultivation at different conditions (Figure 1). A typical process could comprise a large-scale main cultivation in a so-called continuous or fed-batch mode in which at least one substrate is growth limiting, such as in the resveratrol process (Shin et al., 2012). Usually, a number of serial cultivations in batch mode precede the main cultivation step in order to propagate the inoculum. In principle, serial cultivation steps aim at building up biomass while for the final-stage cultivation it is desirable to re-direct the metabolic fluxes such that the product of interest is formed at high yield and rate (Borodina and Nielsen, 2014) (Figure 1). Constraints are therefore very different between the seed train, which prioritizes strain stability, efficient biomass production, process convenience and robustness, and the main cultivation step, often referred to as the “production phase,” where maximal yield and product formation rate are ultimate goals. The bioprocess is therefore imposing a number of constraints on strain design that need to be taken into account early in process development.

In order to better match expression of metabolic pathways to changing conditions in the cells or in the fermentation process, a number of research groups have recently developed strategies for dynamic regulation. As opposed to static up- or down-regulations, dynamic regulation allows for re-balancing fluxes along with changing conditions. Dynamic gene expression profiles allow trade-offs between growth and production to be better managed and can help avoid build-up of undesired intermediates (Brockman and Prather, 2015b). In their review, Brockman and Prather (2015b) provide experimental demonstration of the benefits arising from dynamic control on production of a number of chemicals. Farmer and Liao improved the yield of lycopene production by 18-fold in *E. coli* by implementing a sensor responding to the build-up of acetyl-phosphate (Farmer and Liao, 2000). By modulating protein levels of glucokinase via a genetic inverter, Solomon et al. redirected glucose into gluconate production and improved titers by 30% (Solomon et al., 2012). Using a metabolic toggle switch in *E. coli* to conditionally downregulate citrate synthase expression, Soma et al. redirected acetyl-CoA into isopropanol production with an improvement on titer and yield of more than 3-fold (Soma et al., 2014). Dahl et al. used whole-genome transcript arrays to identify promoters that respond to the accumulation of a toxic intermediate of the isoprenoid biosynthetic pathway, i.e., farnesyl di-phosphate (FPP), and used the identified promoters to regulate FPP formation and improve amorphadiene production two-fold over constitutive or inducible promoters (Dahl et al., 2013). Lo et al. presented a synthetic gene circuit that decouples *E. coli* cell growth from metabolite production through a two-layered circuit based on two genetic sensor controller modules: one sensing feedstock substrate, the other one sensing nutrients (Lo et al., 2016). By delaying enzyme expression until cells have depleted key nutrients and attained high cell densities, the system positively impacted conversion of hydroxycinnamic acids and oleic acid into value-added compounds (Lo et al., 2016). Conditional degradation of essential enzymes has also been exploited to stop elongation of fatty acids and improve octanoate production (Torella et al., 2013), or to increase yield and titer of myo-inositol production (Brockman and Prather, 2015a). Conditional degradation of enzymes is an efficient method for rapidly depleting an enzyme of interest even at slow growth rates where removal via dilution is slow (Brockman and Prather, 2015b).

Dynamic regulation has also been employed in a number of cases in budding yeast. On/off regulation systems like those making use of native repressible promoters like *MET3* promoter, responding to the concentration of methionine, or *HXT1* promoter reacting to glucose concentration were used to redirect the flux in the ergosterol biosynthetic pathway and improve production of isoprenoids of commercial interest (Ro et al., 2006; Asadollahi et al., 2008; Scalcinati et al., 2012). David et al. demonstrated the benefits of hierarchical dynamic control on 3-hydroxypropionic acid (3HP) production (David et al., 2016). Here, a two-layered control with a growth stage control, based on a carbon source responsive promoter, and an intracellular metabolite concentration control, based on a malonyl-CoA sensor, allowed for a sufficient build-up of biomass in the initial growth phase while gradually redirecting metabolism toward 3HP production in the production phase (David et al., 2016). David et al. report a 10-fold increase in 3HP production (David et al., 2016). More recently, dynamic control was used to establish the production of very long chain fatty acid-derived (VLCFA) compounds in *S. cerevisiae*. Production suffered from impaired biomass formation caused by deprivation of essential precursors C_22_-CoA (Yu et al., 2017). Dynamic control, based on carbon-source regulated promoters, was used to relieve the competition between VLCFA product formation and cell-growth associated processes. This strategy divided the process in a production phase and a cell growth phase and resulted in an increase of docosanol production of almost 4-fold. After further optimization, a titer of 83.5 mg/L was achieved which represents a 80-fold improvement compared to the control strain (Yu et al., 2017).

The aim of our study is to increase the number of genetic elements that can be used by metabolic engineers for dynamic control of metabolic pathways. As industrial fermentation processes often comprise different phases (Figure 1), we embarked on identifying and characterizing a set of native yeast promoters that can be used to control expression of metabolic pathways in a dynamic manner, and will positively impact production of value-added chemicals in an industrially relevant context. In order for our tools to be potentially applicable to various kinds of industrial production processes, from bulk to high value-added products, it was decided to use elements already present in the cultivation medium to drive transient expression of genes. Amongst others, glucose is a nutrient often used as a carbon source. Furthermore concentrations of glucose largely differ in the seed train, usually operated as batch, and the final stage fermentation where glucose is often fed in limiting amounts to avoid overflow metabolism. Glucose was thus identified as the element that will trigger the dynamic regulation of gene expression in our system.

Through analysis of genome-wide transcription datasets retrieved from the Gene Expression Omnibus (GEO) database (GEO database), a number of promoters were identified, cloned upstream of a quickly maturing, rapidly degrading green fluorescent protein yEGFP3-Cln2_PEST_ and integrated into *S. cerevisiae* genome. Promoter activity was then characterized in microscale batch and fed-batch conditions. As a proof of the concept, the most promising promoters for transient gene expression were successfully applied to control the production of 3-hydroxypropionic acid.

## Materials and methods

### Analysis of transcriptome data

Transcription data was retrieved from the GEO database, using the R package GEOquery (Davis and Meltzer, 2007), for a microarray study comparing different nutrient limited chemostat cultivations of CEN.PK113-7D (accession GDS777) (Tai et al., 2005). The summarized expression values provided by the authors were log2 transformed before applying a linear model (LIMMA) to identify differentially expressed genes between the glucose-limited experiments and the other nutrient limited experiments for the aerobic series of chemostat cultivations. The differential expression profiles (log2-fold-change of glucose-limited vs. others) for *HXT1, TEF1, ADH1, MAL12* genes were used to find other correlated genes using the Pearson correlation coefficient and test for significance.

### Cloning of the selected promoters upstream of a reporter gene: yEGFP3-Cln2_PEST_ and integration into *S. cerevisiae* genome

Primers for PCR amplification and Uracil excision mediated cloning of the different promoters into the integrative vector pCFB0125 were designed and are listed as Table S1. As the length of promoter regions is not always well defined, the promoter sequence length was either defined based on previous reports or as starting approximately 1,000 bp upstream of the start codon (ATG) when no reference in the literature could be identified. In order to closely follow promoter activity, promoters were cloned upstream of a quickly maturing and rapidly degrading green fluorescent protein encoding gene: yEGFP3-Cln2_PEST_ (Mateus and Avery, 2000). yEGFP3-Cln2_PEST_, as reporter gene, was amplified using the following primers: uGFP_fw: ATCTGTCAU AAA ACA ATGTCTAAAGGTGAAGAATTATTC and uGFP_rv: CACGCGAUTCATATTACTTGGGTATTGCCC) with pCFB0058 (pFA6a-yEGFP3-CLN2-PEST-natMX6, EUROSCARF). Uracil excision was used as cloning method to combine the different promoter-yEGFP3-Cln2_PEST_ expression cassettes together onto pCFB0125. pCFB0125 is a pESC-URA-ccdB vector devoid of its 2micron replication origin, which makes it a non replicative vector that will integrate at the *URA3* locus upon linearization. Standard Uracil excision cloning conditions were used, as described previously (Jensen et al., 2014). After verification of successful cloning, each and every vector was linearized by digestion with restriction enzyme StuI (FastDigest^Ⓡ^, Thermo Scientific) or EcoRV (FastDigestⓇ, Thermo Scientific) in the case of the vector bearing promoter p*MAL32*. Digested, linearized, vectors were transformed into *S. cerevisiae* CEN.PK 113-5D. Transformants were selected on agar plates containing SC-Ura medium and were used for cultivation experiments.

### Construction of 3-hydroxypropionic acid *S. cerevisiae* producing strains

All strains from this study were derived from *S. cerevisiae* CEN.PK102-5B MATa ura3-52 his31 leu2-3/112 MAL2-8c SUC2 obtained from Peter Kötter (Johann Wolfgang Goethe-University Frankfurt, Germany). This strain was further modified by integration of single copy integrative vectors pCFB0380 (PTEF1-SeACS_L641P; PPGK1-ALD6; KlLEU2) and pCFB0382 (PTEF1-PDC1; SpHIS5). The latter strain is the parent strain for the 3HP producing strains of this study. Four Plasmids were constructed based on pCFB0343 (PTEF1-ACC1^**^; PPGK1/CaMCR; KlURA3; insertion site Easyclone X-2). More details about the Easyclone plasmid set and integration sites can be found at Jensen et al. (2014). Backbone vector fragment was prepared for USER cloning by PCR using primers vec_open_mid_ptrTEF1-PGK1 and vec_open_CaMCR and template pCFB0343. Three promoter containing fragments were prepared by PCR using *S. cerevisiae* genomic DNA as template and primers pHXT7_switch and pHXT7_rev for promoter p*HXT7*, pADH2_switch and pADH2_rev for promoter PADH2, and primers pICL1_switch and pICL1_rev for promoter p*ICL1*. Primer sequences can be found in Table 1.

**Table 1:** DNA sequences of PCR primers for USER cloning.

**Promoter**	**Primer**
vec_open_mid	ATGCGAUGCACACACCATAGCTTCAAAATG
ptrTEF1-PGK1	
vec_open_CaMCR	ATCTGTCAUAAAACAATGAGTGGTACAGGTAGATTAGC
pHXT7_switch	ATCGCAUCCGTGGAAATGAGGGGTATGC
pHXT7_rev	ATGACAGAUTTTTTGATTAAAATTAAAAAAACTTTTTGTTTTTG
pADH2_switch	ATCGCAUCGCAGGCGGGAAACCATCCA
pADH2_rev	ATGACAGAUTGTGTATTACGATATAGTTAATAGTTGATAGTTG
pICL1_switch	ATCGCAUTTCCATTCATCCGAGCGATCAC
pICL1_rev	ATGACAGAUTTTTCGTTGACTTTTTGTTATGTTATGC

By combining, under standard uracil exclusion cloning conditions (Jensen et al., 2014), backbone vector prepared as described above and insert PHXT7, PADH2 or PICL1, one obtains three single integrative vectors pCFB0728 (PTEF1-ACC1^**^; PHXT7-CaMCR; insertion site Easyclone X-2), pCFB0727 (PTEF1-ACC1^**^; PADH2-CaMCR; insertion site Easyclone X-2) and pCFB0729 (PTEF1-ACC1^**^; PICL1-CaMCR; insertion site Easyclone X-2), respectively. After transforming strain *S. cerevisiae* CEN.PK102-5B MATa ura3-52 his31 leu2-3/112 MAL2-8c SUC2 X-3::PTEF1-SeACS_L641P; PPGK1-ALD6; KlLEU2 X-4::PTEF1-PDC1; SpHIS5 with integrative vector pCFB0343, pCFB0728, pCFB0727, or pCFB0729, 4 strains are obtained that bear pPGK1-CaMCR, pHXT7-CaMCR, pADH2-CaMCR or pICL1-CaMCR, at integration site X-2 respectively.

### Cultivation

Strains bearing the different promoter-yEGFP3-Cln2_PEST_ expression cassettes were cultivated in two different conditions: (1) in batch mode with mineral medium containing glucose as sole carbon source (Jensen et al., 2014); (2) in fed-batch mode with synthetic fed-batch medium for *S. cerevisiae* M-Sc.syn-1000 purchased from m2p-labs GmbH (Baesweiler, Germany). M-Sc.syn-1000 medium was supplemented with the supplied vitamins solution (final 1% v/v) and the enzyme mix (final concentration 0.5% v/v) immediately prior to use.

Mineral medium contained (L^−1^): 7.5 g (NH4)_2_SO_4_, 14.4 g KH_2_PO_4_, 0.5 g MgSO_4_-7H_2_O, 22 g dextrose, 2 mL trace metals solution, and 1 mL vitamins. The pH of the medium was adjusted to 6 prior to autoclavation. Vitamin solution was added to the medium after autoclavation. Trace metals solution was added after autoclavation. Trace metals solution contained (L^−1^): 4.5 g CaCl_2_-2H_2_O, 4.5 g ZnSO_4_-7H_2_O, 3 g FeSO_4_-7H_2_O, 1 g H_3_BO_3_, 1 g MnCl_2_-4H_2_O, 0.4 g Na_2_MoO_4_-2H_2_O, 0.3 g CoCl_2_-6H_2_O, 0.1 g CuSO_4_-5H_2_O, 0.1 g KI, 15 g EDTA. The trace metals solution was prepared by dissolving all the components except EDTA in 900 mL ultrapure water at pH 6. The solution was then gently heated and EDTA was added. In the end, the pH was adjusted to 4, and the solution volume was adjusted to 1 L and autoclaved (121°C in 20 min). This solution was stored at + 4°C. Vitamin solution contained (L-1): 50 mg biotin, 200 mg p-aminobenzoic acid, 1 g nicotinic acid, 1 g Capantothenate, 1 g pyridoxine-HCl, 1 g thiamine-HCl, 25 g myo-inositol. Biotin was dissolved in 20 mL 0.1 M NaOH and 900 mL water is added. pH was adjusted to 6.5 with HCl and the rest of the vitamins were added. pH was re-adjusted to 6.5 just before and after adding m-inositol. The final volume was adjusted to 1 L, sterile-filtered and stored at + 4°C.

Composition of M-Sc.syn-1000, vitamin solution and enzyme mix are proprietary and were not disclosed to us.

The main difference between the mineral medium and the synthetic M-Sc.syn-1000 used here deals with the carbon source that each contains. In mineral medium used for batch cultivations, glucose is present as at a starting concentration of 20g.L^−1^ while synthetic medium M-Sc.syn-1000, used for fed-batch, contains a solubilized glucose polymer which, due to the activity of glucose releasing enzymes, allows for the slow release of glucose monomers and limits biomass production.

All cultivations were performed at 30°C with 1,000 rpm in the microbioreactor system BioLector (m2p-labs). Forty eight cultivations were run in parallel in FlowerPlates (m2p-labs) with a working volume of 1.1 mL per well. Biomass growth (light scattering units, LSU) and fluorescence (relative fluorescence units, RFU) were monitored online approximately every 12 min.

### Outlier removal and data correction

Some cultivations were removed before further analysis due to data quality or failure of the cultivation to proceed normally.

### Background correction and alignment of measurements

To correct the biomass measurements for background signal, we calculated the growth rates μ via two different methods and compared them for various possible backgrounds to determine the optimal background value. The first method is derived from Poulsen et al. (2003), and is known as the log of slope (LOS) method.

Poulsen et al. (2003) note that experimental data that can be described by an exponential function sometimes contains an offset, giving a function of the form

(1) $$\begin{array}{r} b=b_{0}\cdot exp\left(\mu t\right)+c \end{array}$$

Where *b* is biomass and *t* is time, μ is growth rate, *c* is the background, *b*_0_ is the initial value of of *b* at *t* = 0. In order to remove the background and calculate μ, take the derivative with respect to *t* and take the natural log to get

(2) $$\begin{array}{r} log\left(\frac{db}{dt}\right)=log\left(b_{0}\mu \right)+\mu t \end{array}$$

Thus, the slope of any rectilinear section of the LOS plot will be the specific growth rate μ.

Here, we extend this past what is described by Poulsen et al. (2003) by taking the derivative of Equation (2) with respect to time and further simplifying with the chain rule to get:

(3) $$\begin{array}{r} \mu =\frac{d}{dt}\left(log\left(\frac{db}{dt}\right)\right)=\frac{d^{2}b}{dt}/\frac{db}{dt} \end{array}$$

Thus, we see that if we have a function describing the data, assuming an underlying exponential dependence on *t*, dividing the second derivative of this function by the first derivative will give us μ. This expression for μ is not dependent on the magnitude of *b* and is thus independent of background.

The second method of calculating growth rate is simply the definition of a growth rate normalized to population size:

(4) $$\begin{array}{r} \mu =\frac{db/dt}{b} \end{array}$$

This method is commonly applied for calculating growth rate of cell populations (Ronen et al., 2002; De Jong et al., 2010; Rudge et al., 2016). As this depends directly on *b*, it is thus dependent on background.

It was observed that the biomass profiles of batch cultures exhibit a slight peak at the diauxic shift (Altintas et al., 2016; Figure 3B). For each biomass profile, the time of the peak was identified by determining the time at which light scattering began decreasing. If no peak was found, the peak was artificially set to 10 h for batch cultures and 33 h for fed-batch cultures. A window of 7.5 to 0.5 h before the diauxic shift peak for batch cultures and 20.5 to 1.5 h before the diauxic shift peak for fed-batch cultures was then used to calculate growth rates via each of the two methods described above in Equations (3) and (4). These time windows were chosen as the growth curves appeared approximately exponential in these regions, which is an assumption of the LOS method. Derivatives were computed numerically from a smoothing spline fit to the data. The first and last 6 points of the calculated growth rates were trimmed to account for edge effects of the smoothing spline and the sum of squared errors (SSE) between the remaining growth rate profiles were compared. The growth rate calculation via the Equation (4) was repeated for a range of background values from 0 to 13 for batch cultures and 0 to 10 for fed-batch and compared for each value to growth rate calculated via Equation (3). The background value giving the minimum SSE for each biomass profile was chosen.

To correct for background fluorescence, we used fluorescence measurements from a control strain (p0125) cultivated under the same conditions as experimental strains. These measurements were used to define a function relating control fluorescence (autofluorescence) as a function of biomass, which we will call *f*_*c*_(*b*). For each experimental strain, each biomass measurement was input into *f*_*c*_(*b*) to obtain the autofluorescence for that amount of biomass. This autofluorescence was then subtracted from the corresponding experimental fluorescence associated with that biomass measurement. This process was carried out for all biomass/fluorescence measurements in each experimental strain, resulting in a background-corrected fluorescence profile for each replicate.

In order to compare replicates we aligned the growth curves such that landmark events occur at the same time. This is necessary as the lag phase may last different amounts of time for different cultivation experiments even when the pattern of biomass accumulation is the same after entry to exponential phase. In this case we are interested in the diauxic shift so we used this as the landmark with which to align the growth curves. As previously described we identified the diauxic shift peak and determined the difference between this landmark and a reference time, which we set to be at 15 h for batch cultures and 30 h for fed-batch cultures. This difference was subtracted from the entire set of times corresponding to both biomass and fluorescence measurements to determine the corrected times; moving forward, we only use data from corrected times greater than 0. A similar procedure for growth curve alignment is discussed by van Ditmarsch and Xavier (2011).

### Promoter activities in small-scale batch cultivations

Background-corrected biomass and fluorescence profiles were input into the PromAct model (Kannan et al., 2018) using a protein maturation rate of 0.45 *h*^−1^ as described for EGFP (Heim et al., 1995) and a protein degradation rate of 0.5 *h*^−1^ as described for yEGFP3-Cln2_PEST_ (Mateus and Avery, 2000). mRNA degradation rate was set as $\frac{1}{3}\;h^{-1}$ based on genome-wide estimates of mRNA degradation in *S. cerevisiae* (Munchel et al., 2011; Neymotin et al., 2014).

The promoter activity profiles were cropped to 35 h for batch and 60 h for fed-batch cultures on the corrected time scale for standardization. Lag phase was identified as when the rate of change of the average biomass profile over all replicates from a given promoter first surpassed 0.5 LSU per h. The diauxic shift was identified as the time between the characteristic peak, identified as where the derivative of the average biomass profile over all replicates becomes crosses 0, to when the derivative next surpasses 0.5 LSU per h following this peak. For batch cultures, the stationary phase was also identified using the same peak detection method as with the diauxic shift identification, as a similar characteristic peak is seen. A similar method for growth phase identification was used by Altintas et al. (2016). “Early” and “Late” designations in Figure 4 indicate the first and second half of each growth phase, respectively.

### Measurement of 3-hydroxipropionic acid concentration

Strains expressing CaMCR (malonyl-CoA reductase from *Chloroflexus aurantiacus*) for the production of 3HP were cultivated in FlowerPlates in the BioLector, in the same conditions as described above, in batch and fedbatch modes. Samples for 3HP measurement were taken during the course of the cultivations, 3HP concentration was measured using a dedicated enzyme assay. The enzyme assay was performed as described in the supplementary materials of Borodina et al. (2015).

## Results

### Selection of promoters through analysis of genome-wide transcription datasets

In order to identify promoters with glucose-dependent expression profiles, we searched the literature for genome-wide expression datasets comparing glucose-limited and glucose excess conditions. Boer et al. (2003) characterized the specific transcriptional responses of *S. cerevisiae* cells growing at steady state in chemostats under growth limitation by carbon, nitrogen, phosphorus, or sulfur. We performed an analysis of this dataset in order to directly compare gene expression levels in the glucose limiting condition to the average of expression levels observed while not glucose limited (e.g., combining the results from the sulfur, the phosphorus and the nitrogen limiting conditions) which we describe as “glucose excess” conditions.

**Figure 2:**
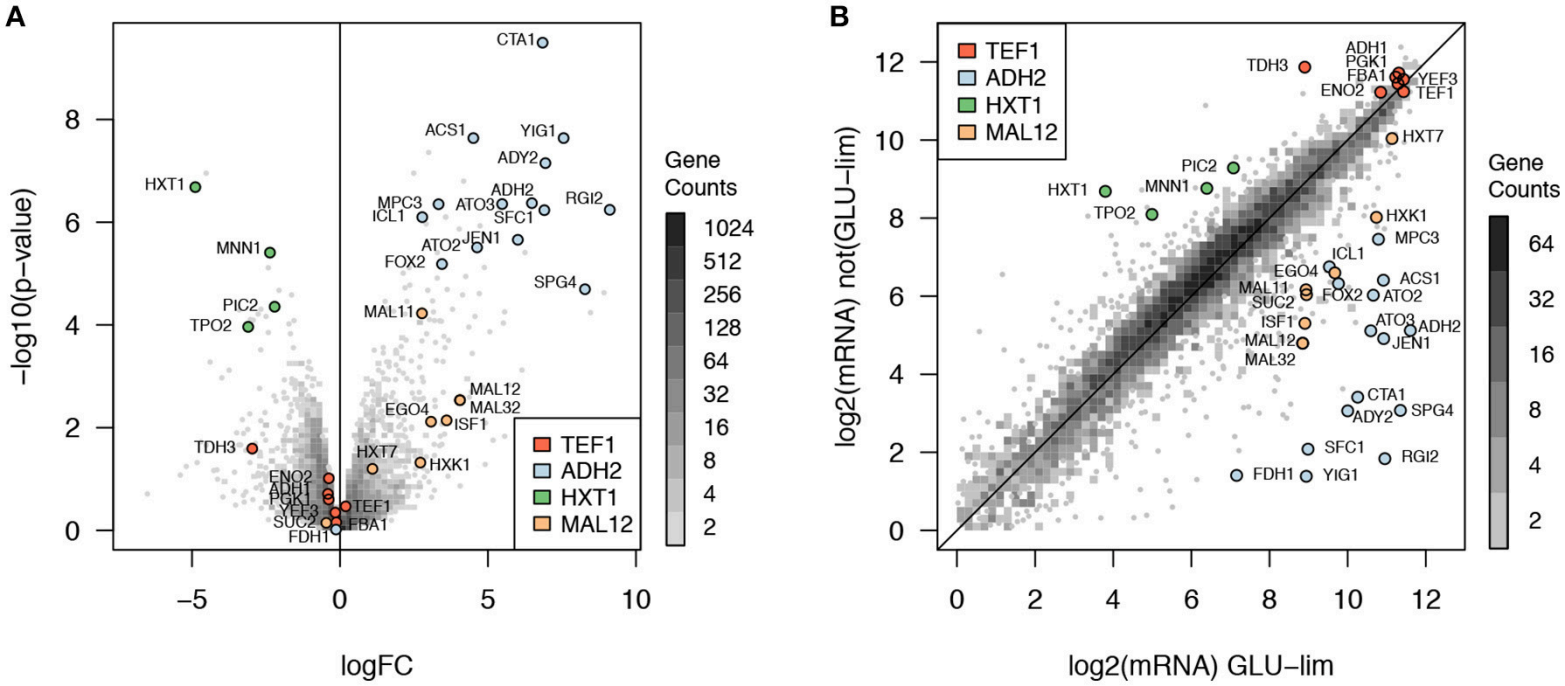
Comparison of expression levels between glucose excess and glucose limiting cultivation conditions. **(A)** Volcano plot with a 2D histogram of gene counts, **(B)** plot of expression levels in glucose limiting vs. glucose excess conditions with a 2D histogram of gene counts. The four gene expression archetypes identified are represented: (1) *TEF1* archetype (red) with constitutive expression in both conditions, (2) *HXT1* archetype (green) with high expression in glucose-excess and low expression in glucose-limiting conditions, (3) *ADH2* (light blue), and (4) *MAL12* (orange) archetypes with low expression in glucose-excess and high expression in glucose limiting conditions.

The 2D histogram (Figure 2B) displays the comparison of gene expression levels between glucose-limited and glucose excess conditions. The volcano plot (Figure 2A) displays the genes found significantly differently expressed between glucose-limited and glucose excess conditions. Four archetypes of gene expression were identified for their known roles and are represented in Figure 2: (1) *TEF1* archetype with constitutive expression in both conditions, (2) *HXT1* archetype with high expression in glucose-excess and low expression in glucose-limiting conditions, (3) *ADH2*, and (4) *MAL12* archetypes with low expression in glucose-excess and high expression in glucose limiting conditions. As some of the archetypes are not differentially expressed in this comparison, e.g., *TEF1*, the identified genes correlated with them are most often not differentially expressed in this comparison. By contrast, genes significantly correlated to *ADH2* and *HXT1* were among the most differentially expressed in glucose-excess vs. glucose limiting conditions.

From the archetype correlation analysis, 34 genes were identified with diverse expression profiles in respect to dynamic regulation of gene expression. Promoter regions from all 34 genes were cloned (or attempted) as reported in the materials and methods section. Although not all were expected to be significantly differentially expressed, 20 genes were found significantly up-regulated in glucose limiting conditions and 5 genes were significantly down-regulated in glucose excess conditions (*p* < 0.05 after Benjamini-Hochberg correction).

### Characterization of promoter activities in microfermentations

#### Microscale batch cultivations

**Figure 3:**
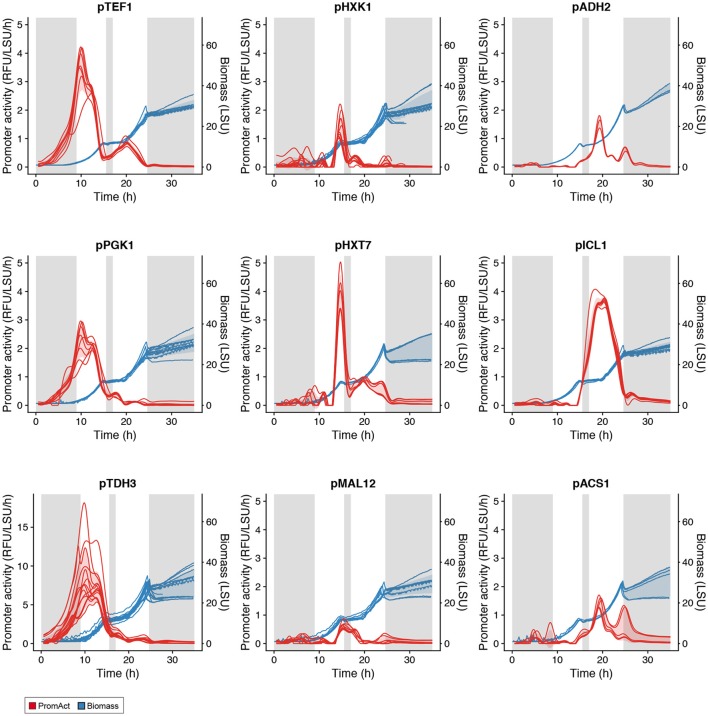
Activity of various promoters in batch cultivations. Gray shaded areas are low-growth phases. Red shading represents one standard deviation from the mean promoter activity over all replicates at each time point (see Table S2). Blue shading represents one standard deviation from the mean biomass value over all replicates at each time point.

Batch cultivations were followed until stationary phase was reached. As presented in Figure 3, the two classical growth phases of a *S. cerevisiae* batch fermentation, where glucose is the sole carbon source, were clearly observed with a glucose growth phase followed by an ethanol growth phase separated by a diauxic shift (Monod, 1941). A few typical profiles of promoter activity are presented in Figure 3: constitutive expression, i.e., *TEF1*, high expression in glucose-excess conditions, i.e., *PGK1* and *TDH3*, activation at low glucose concentration, i.e., *HXK1, HXT7*, and *MAL12* or expression when growing on ethanol in the absence of glucose, i.e., *ADH2, ICL1*, and *ACS1*. None of the promoters tested showed sustained activity in stationary phase, likely due to the lack of metabolic activity in the absence of carbon sources. Promoter activities of other promoters tested can be found in Figure S1.

**Figure 4:**
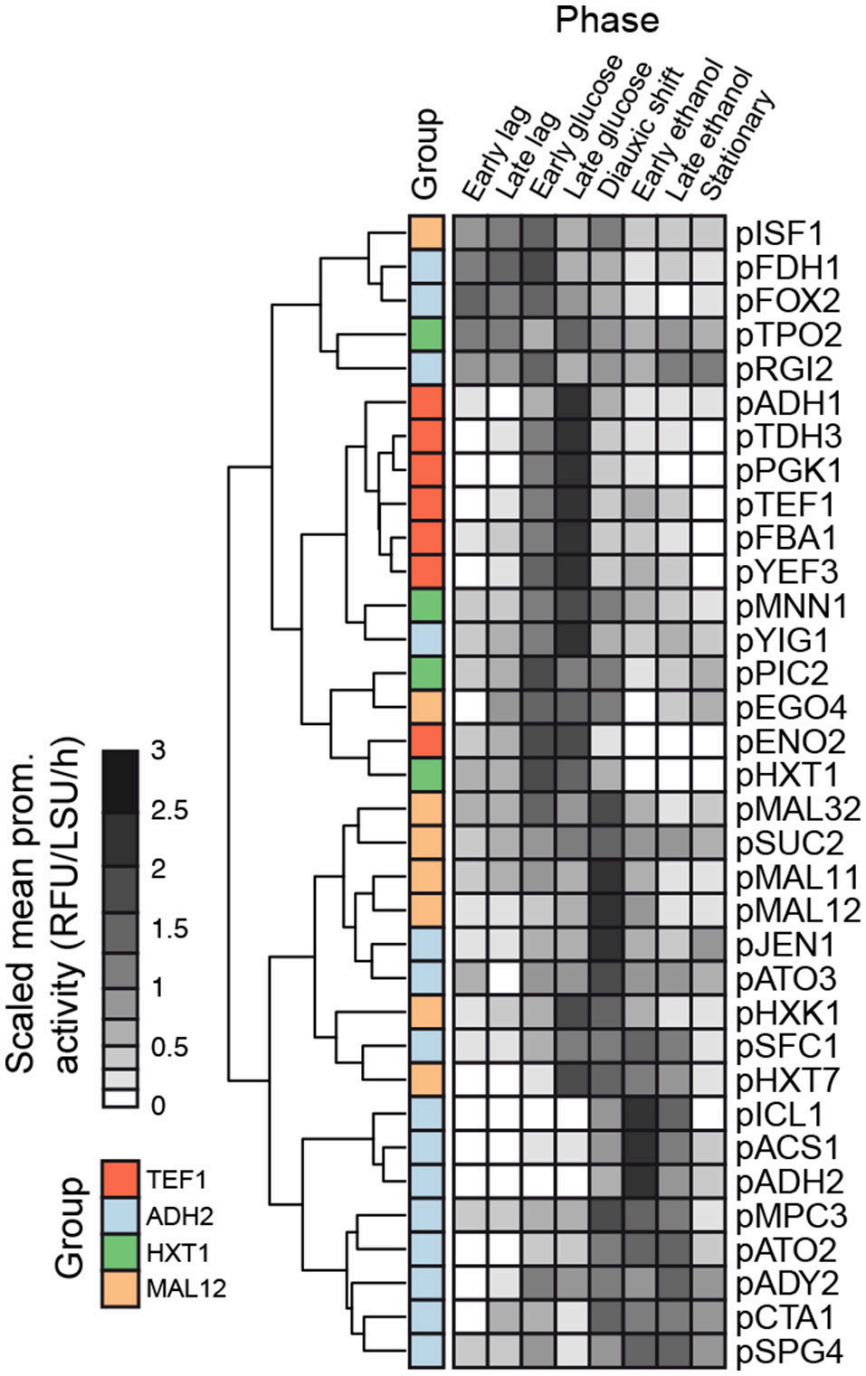
Comparison of promoter activities. Clustering of dynamic promoter activity profiles summarized by the average activity in 8 non-overlapping time windows representing the growth phases in batch cultivations. Time windows were defined as follows: Early lag 0.50–1.50, Late lag 1.50–3.00, Early glucose 3.00–9.25, Late glucose 9.25–15.5, Diauxic shift 15.5–17.0, Early ethanol 17.0–20.8, Late ethanol 20.8–24.6, Stationary 24.6–35.0.

A heat map and cluster analysis were applied to all promoter activities in the batch condition (Figure 4). The cluster analysis reveals a tight correlation between promoter activities experimentally determined here and the gene expression categories defined from the analysis of genome-wide transcription data presented in Figure 2, which reflects that the different promoters cloned upstream of yEGFP3-Cln2_PEST_ respond mostly in a similar fashion as when they are in their native context. Globally, two main branches appear, at the top of the figure promoters active in glucose excess conditions, and at the bottom can be observed promoters activating at low glucose concentration or when glucose is absent (Figure 4). This cluster analysis grouped profiles by archetype group in all but 6 cases where cluster grouping seemed to contradict with the gene expression data. This was observed for the promoters of *ISF1, FDH1, FOX2, RGI2, YIG1*, and *EGO4* (Figure 4). In the case of *EGO4*, (*RGI2 YIL057C*), and *FOX2*, this may be explained by their unclear promoter activity patterns (Figure S1). The three others, *ISF1, FDH1*, and *YIG1*, clearly behave in opposite fashion compared to what was expected from the gene expression analysis (Figure 2).

#### Microscale fed-batch cultivations

**Figure 5:**
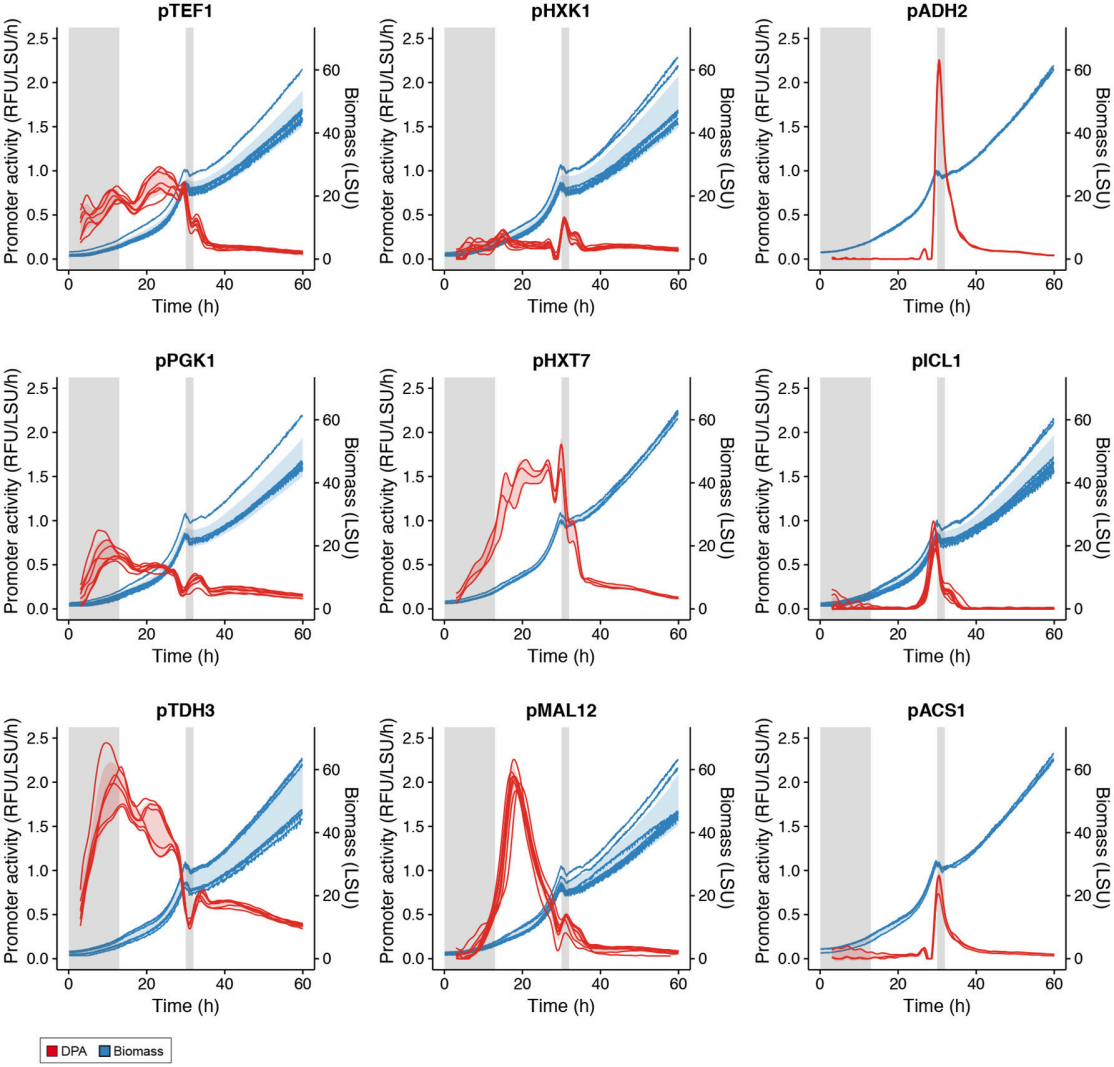
Activity of various promoters in fed-batch cultivations. Gray shaded areas are low-growth phases. Red shading represents one standard deviation from the mean activity over all replicates (see Table S3). Blue shading represents one standard deviation from the mean biomass value over all replicates at each time point.

In this microscale fed-batch cultivation, realized using the glucose slow release technology Feed-In-Time (m2p-labs), two main growth phases are observed: (1) an initial phase until approximately 25 h of cultivation where residual glucose is still present at a low level (2) a glucose-limiting growth phase after 25 h of cultivation where biomass growth is limited by the slow release of glucose (Figure 5).

In terms of promoter activity, two major activity profiles appear: (1) promoters characterized by a higher and sustained activity in the first growth phase that remain active in the second phase, although to a lower extent (e.g., *TEF1, PGK1, TDH3, HXT7, MAL12*); and (2) promoters that strongly activate at the onset of glucose limiting phase and that quickly become at most weakly active or inactive (*ADH2, ACS1, ICL1*).

Based on the outcome of promoter activity characterization, it was decided to run a proof of concept study and test a subset of promoters for transient expression and controlled production of a commercially relevant compound, with the objective of restricting its production only in “production” phase in fed-batch and not during the seed train phases carried on as batch.

### Proof of concept study: dynamic regulation of 3-hydroxypropionic acid (3HP) production

3HP is a platform chemical that can be converted into acrylic acid, 1,3-propanediol, malonic acid, and other valuable chemicals. In 2011, the world annual production of acrylic acid was 5000 kMT and the market size was USD 11.5 billion (Borodina et al., 2015). Acrylic acid-derived products include superabsorbent polymers used in diapers and incontinence products, plastics, coatings, adhesives, elastomers, and paints (Borodina et al., 2015). In biological systems, 3HP can be synthesized via at least four different intermediates: glycerol, lactate, malonyl-CoA or β-alanine (Kumar et al., 2013). Production of 3HP based on the β-alanine or the malonyl-CoA have been reported in *S. cerevisiae* (Chen et al., 2014; Borodina et al., 2015; Kildegaard et al., 2016). Furthermore, Kildegaard et al. reported a reduced growth rate of the strains expressing the malonyl-CoA route for 3HP production, especially in the case where pathway genes were integrated in multiple copies (Kildegaard et al., 2016).

**Figure 6:**
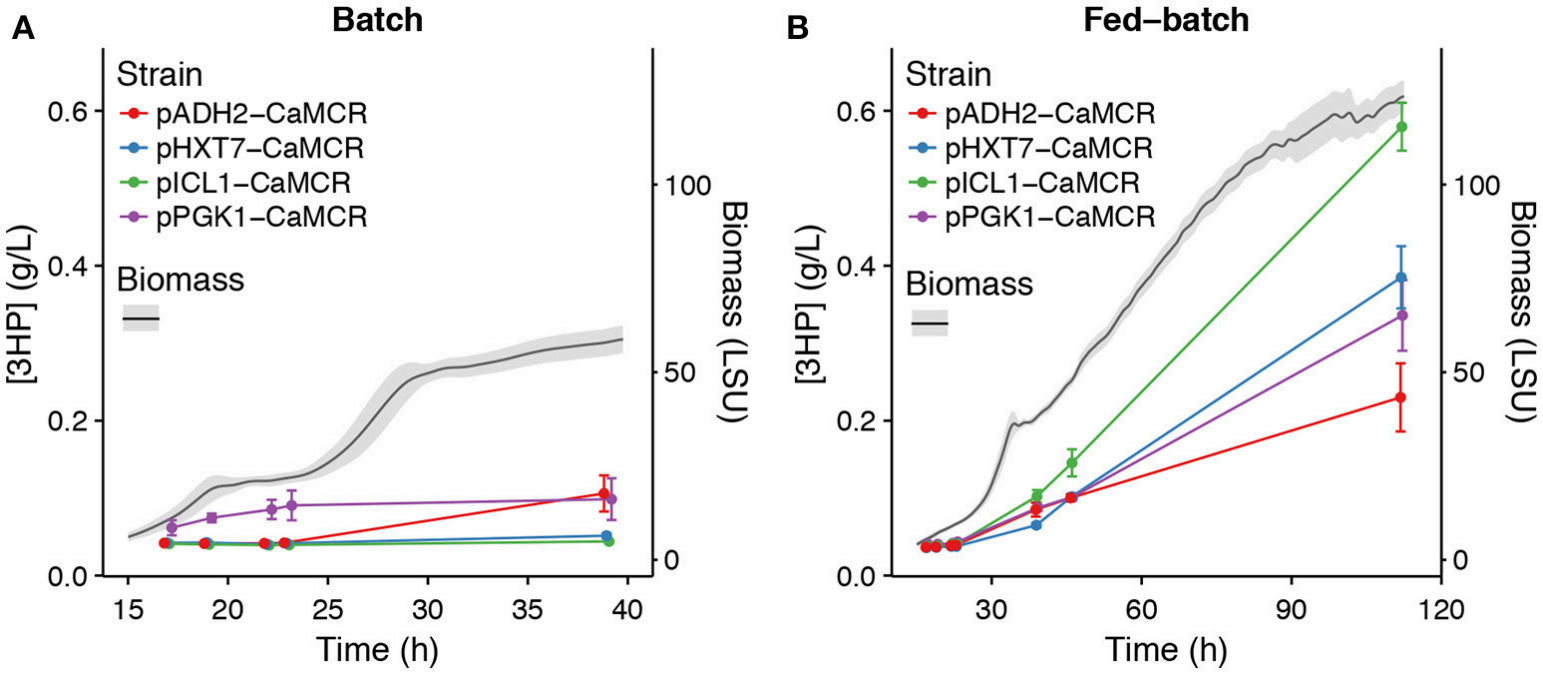
Effect of dynamic control of gene expression on 3HP production. 3HP production in **A**: batch and **B**: fed-batch cultivations for p*PGK1*, p*HXT7*, p*ICL1*, or p*ADH2* driving production of CaMCR. Error bars on 3HP levels represent standard error of the mean, *n* = 3. The gray ribbon on Biomass represents plus/minus one standard deviation. 3HP concentration is in g/L and Biomass is measured in light scattering units (LSU).

In an attempt to control production of 3HP via the malonyl-CoA route, with the objective to drive 3HP production only in “production” phase in fed-batch and not during the seed train phases carried on as batch, three promoters were selected based on their expression profiles: p*HXT7*, p*ADH2*, and p*ICL1*. They were selected for their weak (if not absent) expression in cultivation with excess of glucose and their induction in glucose limiting conditions (Figure 3). Promoters of *HXT7, ADH2*, and *ICL1* were then cloned upstream of the gene encoding bi-functional malonyl-CoA reductase from *Chloroflexus aurantiacus* (CaMCR), which is the only heterologous enzyme necessary for producing 3HP via the malonyl-CoA route in *S. cerevisiae*. As a reference, CaMCR was expressed under the control of p*PGK1*, a promoter which is both active in glucose excess and glucose limiting conditions (Figures 3, 5). When p*PGK1* is controlling the expression of CaMCR, a clear accumulation of 3HP is observed already at the early stages of growth on glucose in the batch condition (Figure 6). This is clearly not the case when the (glucose-) regulated promoters p*HXT7*, p*ADH2* and p*ICL1* are controlling CaMCR expression, as here production of 3HP remains below detection limit of the assay (Figure 6). In the fed-batch cultivation condition, where all promoters are being activated, 3HP production is restored. Expressing CaMCR from the regulated *ICL1* promoter resulted in a 1.7-fold increase in 3HP production when compared to the constitutive expression from *PGK1* promoter (Figure 6).

It is clearly demonstrated here that production of a metabolite of interest can be turned on and off by using a set of dynamically activated promoters. Furthermore, turning off 3HP production in growth phases of batch fermentation did not affect the final production titer in the fed-batch condition, and was even beneficial, especially when p*ICL1* controlled expression of CaMCR.

## Discussion

In this study, specific genome-wide expression datasets were analyzed in order to identify genes characterized by different expression patterns in glucose excess as compared to glucose limiting conditions. Four archetypes of gene expression were defined. A number of genes with expression patterns qualified as interesting were selected and their promoters cloned upstream of a quickly maturing rapidly degrading GFP. Destabilized GFP variants are better suited for the study of transient gene expression as native GFP variants have particularly long half-lives. In this study, it was decided to use yEGFP3-Cln2_PEST_ which is a destabilized variant of GFP that has successfully been used to monitor dynamic changes in gene expression in yeast (Mateus and Avery, 2000). After characterizing promoter activities both in batch with glucose as sole carbon source and glucose-limiting fedbatch conditions, a hand full of promoters were chosen for a proof of concept study where controlled production of the commercially relevant chemical 3HP was demonstrated.

Comparing results from the genome-wide gene expression analysis and from the characterization of promoter activity in the batch cultivation conditions, it was interesting to notice the correlation between the archetypes defined for the genome-wide gene expression analysis (Figure 2) and the clusters obtained for the promoter activity characterization (Figure 4). This confirmed that most of the promoters cloned upstream yEGFP3-Cln2_PEST_ respond according to the archetype defined for the gene they relate to. It also indicated that elements responsible for transcriptional regulation of those genes are present in the promoter sequence used in this study. This validated the promoter sequences that were chosen here. Discrepancies were observed though for six genes. For three of them, *ISF1, FDH1*, and *YIG1*, the promoter that was cloned triggered an expression pattern opposite to what was reported for the genome-wide gene expression analysis. This may indicate that the chosen promoter sequence is lacking regulatory elements otherwise present in the native promoter, or that DNA structure at the integration site affects activity pattern of these promoters.

Taking a look at promoter activities measured during the microscale fedbatch cultivation, it appeared that while some promoters were only activated at the onset of glucose limitation (e.g., promoters of *ACS1, ADH2, ICL1, SFC1, SPG4*), others were characterized by a much wider expression time window, with an early activation (e.g., promoters of *HXT7, MAL12, FMP3*) (Figure 5). This may be explained by the fact that the latter promoters are active already in the phase where residual glucose is present but at a low concentration. This early phase of the microscale fedbatch experiment is an intermediary phase caused by the technology employed here, i.e., the Feed-In-Time technology as a low amount of free glucose is initially present in the medium and acts as a carbon source until the slow enzymatic release of glucose from the polymer takes over and triggers glucose limitation. The low concentration of glucose in this early phase may still allow for activation of the above-mentioned promoters (Figure 5).

Surprisingly, promoters like *ADH2, ICL1*, or *ACS1* were characterized by a burst of expression at the onset of the glucose limiting phase. These promoter activity profiles suggest a rapid increase and decrease in their activity although they appear to maintain some activity for roughly 10 h after activation. This was not necessarily expected as the corresponding genes were clearly maintaining expression in the glucose limited continuous cultivation of the genome-wide gene expression analysis (Figure 2). It further underlines the necessity for testing genetic elements for controlled expression in conditions as close as possible to the intended bioprocess.

Benefits of dynamic control of metabolic pathways have been demonstrated before, as described in the introduction. Nevertheless, this study further confirms that transient activation of a metabolic pathway does not negatively impact production of a compound of interest, here 3HP, but rather benefits production performance, as shown here in the case where *ICL1* promoter restricts expression of CaMCR to the microscale fedbatch cultivation stage (Figure 6). Even-though other factors, e.g., timing of MCR expression, substrate availability or pathway intermediate concentrations may also contribute to the benefits observed on 3HP production, it is surprising that such a sudden and short expression timespan triggered by *ICL1* promoter can result in superior production of the compound of interest as compared to longer expression timespan as in the case of p*HXT7* or p*PGK1*. This certainly raises the question whether sudden, short but high expression bursts at the onset of changing conditions could not be sufficient to sustain efficient production of chemicals of interest. Reducing the timespan of expression of a promoter may be beneficial to the overall production process as it may restrict the loads of energy spent on transcription and translation of the specific gene only to a brief time-window. Recently Christiano et al. measured 8.8–12 h as median half lives of proteins in *S. cerevisiae* (Christiano et al., 2014). Therefore, once synthesized the functional protein would still remain in the cells even though the promoter of its gene, hence transcription and translation of that gene, would be off during most of the cultivation. Promoters like *ICL1, ADH2, ACS1*, combined with other synthetic biology tools that could expand mRNA half-lives, e.g., sequences improving mRNA stability (Curran et al., 2013; Yamanishi et al., 2013), may certainly play an active role.

## Conclusion

Through analysis of gene expression data, promoters showing specific responses to glucose excess or limitation were identified. DNA sequences of these promoters were cloned upstream of a quickly maturing and rapidly degrading green fluorescent protein to allow the study of promoter activities. Dynamics of promoter activities for a number of promoters in batch with glucose as sole carbon source, or glucose limiting conditions were analyzed. A subset of promoters, p*ADH2*, p*ICL1*, and p*HXT7*, were demonstrated as suitable for dynamic control of production of a commercially relevant compound, i.e., 3HP, in *S. cerevisiae*. The set of promoters and the dynamic promoter activity profiling presented here will contribute to the molecular biology toolbox available to metabolic engineers.

## Author contributions

IB and JM conceived the design of the study. JN contributed with initial ideas about the project and discussions on the possible promoter set for evaluation. JM, NJ, FÖ, and KK performed DNA work. JM generated the strains and performed small scale cultivations. JM and CW performed the transcriptome data analysis. JF participated in the design of the study and interpretation of initial results. SK and CW performed dynamic promoter activity analyses. SK, CW, and JM analyzed the data. SK, JM, IB, and CW drafted the manuscript and all authors contributed to preparing the final version of the manuscript.

### Conflict of interest statement

The authors declare that the research was conducted in the absence of any commercial or financial relationships that could be construed as a potential conflict of interest.
