# Supplementary material for: Glucose-Dependent Promoters for Dynamic Regulation of Metabolic Pathways
Source: Front Bioeng Biotechnol. 2018 May 22;6:63. doi: 10.3389/fbioe.2018.00063 (PMC5972318; doi:10.3389/fbioe.2018.00063)
Supplement: Supplementary file 1 [file Presentation_1.PDF]

Supplementary Material: Industrial strain design: characterizing promoters for dynamic regulation of metabolic pathways

Jérôme Maury , Soumya Kannan , Niels B. Jensen , Frederik Öberg , Kanchana R. Kildegaard , Jochen Förster , Jens Nielsen , Christopher T. Workman * and Irina Borodina *

*Correspondence:
Christopher T. Workman, Irina Borodina
cwor@dtu.dk, irbo@biosustain.dtu.dk

1 SUPPLEMENTARY DATA

2 SUPPLEMENTARY TABLES AND FIGURES

2.1 Tables

S1 Table.

Table S1 Primers for promoter amplification.

Promoter	Dir.	Primer Sequence
pJEN1	fwd	CGTGCGAUTCCTTTTCTTCGTCATTTCAATGTTATAATC
pJEN1	rev	ATGACAGAUATTTTCAGTAGCAGTATTTAATCTCTTTG
pMAL11	fwd	CGTGCGAUTTATGTAATTTAGTTACGCTTGACTGATG
pMAL11	rev	ATGACAGAUATTATACTATTTTTTTAGTTGTTTGATGTTCTTC
pMAL32	fwd	CGTGCGAUAGTTAATTAATAGTCTTGGATGTAATTCTTATTG
pMAL32	rev	ATGACAGAUTTATGTATTTTAGTTACGCTTGACTGATG
pHXT1	fwd	CGTGCGAUTGCAGGTCTCATCTGGAATATAATTC
pHXT1	rev	ATGACAGAUGATTTTACGTATATCAACTAGTTGACG
pHXK1	fwd	CGTGCGAUTGGCGTGGGGTGGGGTGATTATC
pHXK1	rev	ATGACAGAUCTTATTTTTTCAGTATTCTAATTGAGTTGTTTG

Continued on next page

Table S1 – *Continued from previous page*

Promoter	Dir.	Primer Sequence
pFOX2	fwd	CGTGCGAUTATCCGTACTAGAACTCTCGGC
pFOX2	rev	ATGACAGAUGGCTTACTCAAATAAGAAATAAATCGAATG
pICL1	fwd	CGTGCGAUTTCCATTCATCCGAGCGATCAC
pICL1	rev	ATGACAGAUTTTTCGTTGACTTTTTGTTATGTTATGC
pFMP3	fwd	CGTGCGAUTGTTTCACAAACACGCCTCTATATC
pFMP3	rev	ATGACAGAUAAAAATATAGGTTTGTGTTTCTATGTGTCT
pFDH1	fwd	CGTGCGAUGCAAGTGACATGAACACTGTAATACATC
pFDH1	rev	ATGACAGAUATTAATTTTCAGCTGTTATTTTGATTTG
pFBA1	fwd	CGTGCGAUATAACAATACTGACAGTACTAAATAATTGCCTAC
pFBA1	rev	ATGACAGAUTTTGAATATGTATTACTTGGTTATGGTTA
pENO2	fwd	CGTGCGAUCGCTCAGCATCTGCTTCTTC
pENO2	rev	ATGACAGAUTATTATTGTATGTTATAGTATTAGTTGCTTG
pISF1	fwd	CGTGCGAUAAGTTCTCTTACCCCTTTTTATTTGGCA
pISF1	rev	ATGACAGAUCAAAATACAGCGACTATTTCTATTCT
pCTA1	fwd	CGTGCGAUTAGCCGCGCAAGTTGGTG
pCTA1	rev	ATGACAGAUCTTCTAGGGTTCCAAATTTATTTG
pATO3	fwd	CGTGCGAUCCAAAAGCCTACTGCATTGTAATAC
pATO3	rev	ATGACAGAUTTTAACCAGTATTTTTATTTTTTATTCTTACTAAATCT
pATO2	fwd	CGTGCGAUAGTTCTTGACTACCCCTATCTCAC
pATO2	rev	ATGACAGAUGCCTTTTATTTTTTTCTGTGATTGAAG
pACS1	fwd	CGTGCGAUGGCTATATAATCTTTTTATCACGTCA
pACS1	rev	ATGACAGAUAGCACAGTGGGCAATGTCTTTC
pADH1	fwd	CGTGCGAUGGGTGTACAATATGGACTTCCTC
pADH1	rev	ATGACAGAUTGTATATGAGATAGTTGATTGTATGCT
pADH2	fwd	CGTGCGAUCGCAGGCGGGAAACCATCCA
pADH2	rev	ATGACAGAUTGTGTATTACGATATAGTTAATAGTTGATAGTTG
pADY2	fwd	CGTGCGAUATAGGCGTCGTATATAGTCTCTTC
pADY2	rev	ATGACAGAUATTATTTGTTTGTATATGAGTTGTTTTGTG
pMNN1	fwd	CGTGCGAUTAACGTAAATGCATATCCTGCATCAAG
pMNN1	rev	ATGACAGAUTTAATACACAGTAACACTTTCTTAAACTAGC
pPIC2	fwd	CGTGCGAUGTTCGTAATTATGTATTTTTGCTATTGTATATG
pPIC2	rev	ATGACAGAUGCTTTACAACTGTCAATGAGTCCGT
pPYK1	fwd	CGTGCGAUGATCCAAATGTAAATAAACAATCACAAGG

Continued on next page

Table S1 – *Continued from previous page*

**Promoter**	**Dir.**	**Primer Sequence**
pPYK1	rev	ATGACAGAUTGTGATGATGTTTTATTTGTTTTGATTGG
pSFC1	fwd	CGTGCGAUCCGGTGGTGCTTATACTGTTTC
pSFC1	rev	ATGACAGAUTTTTCTTTCTTCTTCTTTGTTATGTTATC
pSPG4	fwd	CGTGCGAUAGGAGTCTTATGTGACAGCACT
pSPG4	rev	ATGACAGAUATTTGATATCTCGATTAGTTGGTTTTATTTATC
pSUC2	fwd	CGTGCGAUGCTCCCCCAGCAAAGCTC
pSUC2	rev	ATGACAGAUCATATACGTTAGTGAAAAGAAAAGC
pSUC4	fwd	CGTGCGAUTTGTAGCGGGAAGGGTGCT
pSUC4	rev	ATGACAGAUCATATACGTTAGTGAAAGGAAAAGC
pTDH3	fwd	CGTGCGAUATAAAAAACACGCTTTTTCAGTTCG
pTDH3	rev	ATGACAGAUTTTGTTTGTTTATGTGTGTTTATTCGA
pTPI1	fwd	CGTGCGAUCTACTTATTCCCTTCGAGATTATATCTAG
pTPI1	rev	ATGACAGAUTTTTAGTTTATGTATGTGTTTTTTGTAGTTATAG
pTPO2	fwd	CGTGCGAUGTACCTATGCAAAAACCCTTCC
pTPO2	rev	ATGACAGAUATTTGTTTTGTGTATTATTTTTGTGATTAG
pYEF3	fwd	CGTGCGAUGCAACTTTCCCTTCTGTTTCAATC
pYEF3	rev	ATGACAGAUCTTTTAATGTTATCGATGGATTCGAAC
pYIG1	fwd	CGTGCGAUTTTCTAGTTCTTCTTCTGCAATATTG
pYIG1	rev	ATGACAGAUGATTTCGTTATGATTATTTTCTTTCTTGAG
pYIL057C	fwd	CGTGCGAUGCGCAGAATTCAGGGCAAAAC
pYIL057C	rev	ATGACAGAUTCTTATTATTTAAAATACTTTGTTATATACTGTAC
pMAL12	fwd	CGTGCGAUATTATACTATTTTTTTAGTTGTTTGATGTTCTTC
pMAL12	rev	ATGACAGAUTTATGTAATTTAGTTACGCTTGACTGATG
pPGK1	fwd	CGTGCGAUGAAGTACCTTCAAAGAATGGGGTC
pPGK1	rev	ATGACAGAUTTGTTTTATATTTGTTGTAAAAAGTAGATAATTACTTC
pTEF1	fwd	CGTGCGAUGCACACACCATAGCTTCAAAATG
pTEF1	rev	ATGACAGAUTTGTAATTAAAACTTAGATTAGATTGCTATG
pHXT7	fwd	CGTGCGAUCCGTGGAAATGAGGGGTATGC
pHXT7	rev	ATGACAGAUTTTTTGATTAAAATTAAAAAAACTTTTTGTTTTTG
pYNR034	fwd	CGTGCGAUATATTTGTATATTATTAGATATGTATGCAAAC
pYNR034	rev	ATGACAGAUCTTGCAAAAAATTATTCCAGTTAAAATC

Note: YNR034 is to amplify the promoter of YNR034W-A

S2 Table.

Number of included replicates per promoter in batch cultivations.

Table S2 Replicates by promoter.

Promoter	$n_{replicates}$	n_{plates}	Promoter	$n_{replicates}$	n_{plates}
pACS1	5	2	pJEN1	6	1
pADH1	3	1	pMAL11	3	1
pADH2	3	1	pMAL12	9	3
pADY2	3	1	pMAL32	6	2
pATO2	4	2	pMNN1	6	2
pATO3	3	1	pPGK1	8	3
pCTA1	3	1	pPIC2	3	1
pENO2	3	1	pSFC1	8	3
pFBA1	7	3	pSPG4	4	2
pFDH1	3	1	pSUC2	3	1
pFMP3	9	3	pTDH3	14	5
pFOX2	3	1	pTEF1	7	2
pHXK1	11	3	pTPO2	3	1
pHXT1	3	1	pYEF3	9	4
pHXT7	6	2	pYIG1	3	1
pICL1	14	4	pYIL057C	3	1
pISF1	3	1	pYNR034	3	1

S3 Table.

Number of included replicates per promoter in fed-batch cultivations.

Table S3 Replicates by promoter.

Promoter	$n_{replicates}$	n_{plates}	Promoter	$n_{replicates}$	n_{plates}
pACS1	3	1	pMAL12	8	2
pADH2	3	1	pPGK1	7	2
pFBA1	3	1	pSFC1	3	1
pFMP3	3	1	pSPG4	7	2
pHXK1	8	2	pTDH3	6	2

Continued on next page

Table S3 – *Continued from previous page*

Promoter	$n_{replicates}$	n_{plates}	Promoter	$n_{replicates}$	n_{plates}
pHXT7	3	1	pTEF1	7	2
pICL1	9	2	pYEF3	4	2

2.2 Figures

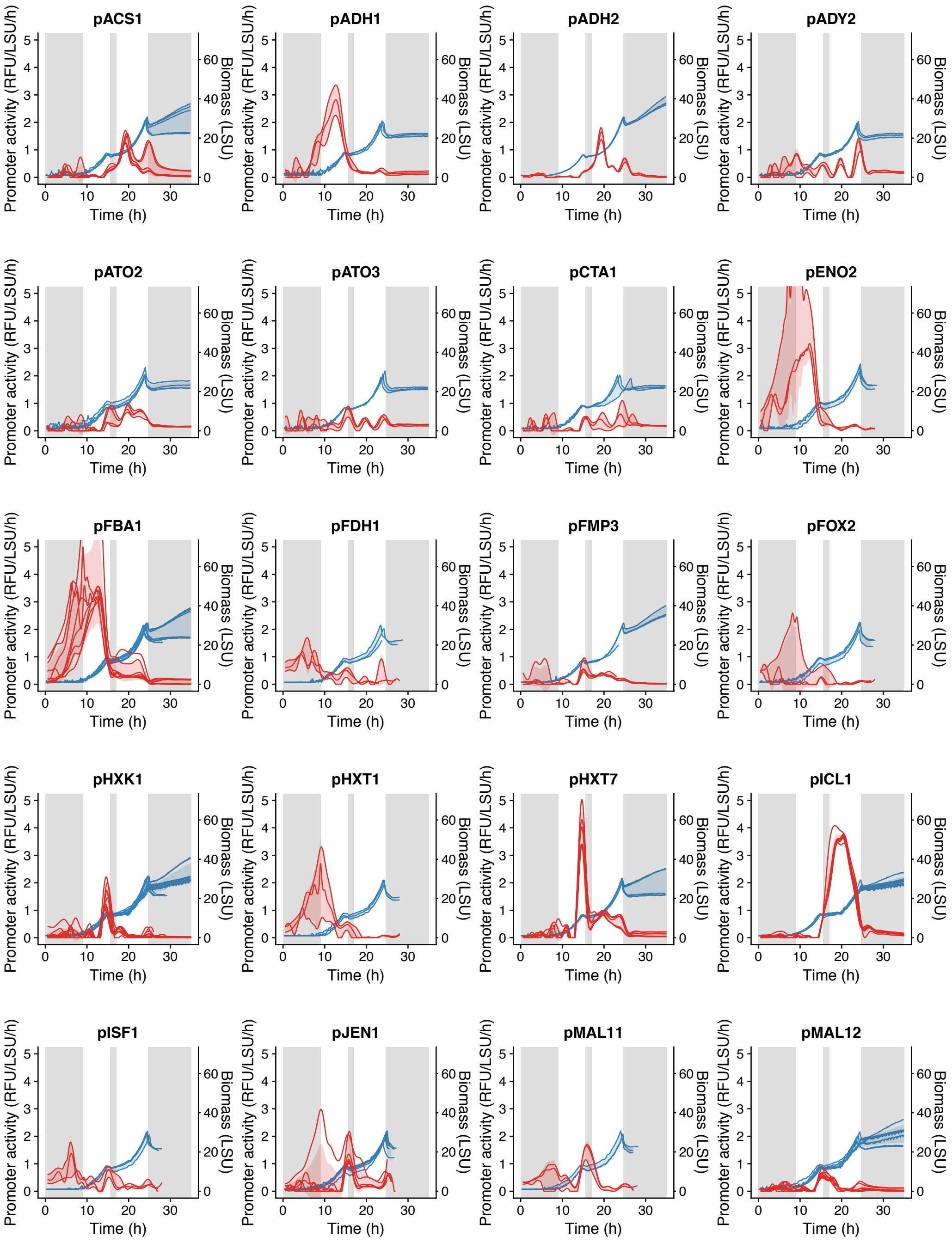
pACS1
pADH1
pADH2
pADY2
pATO2
pATO3
pCTA1
pENO2
pFBA1
pFDH1
pFMP3
pFOX2
pHXK1
pHXT1
pHXT7
pICL1
pISF1
pJEN1
pMAL11
pMAL12
Promoter activity (RFU/LSU/h)
Biomass (LSU)
Time (h)

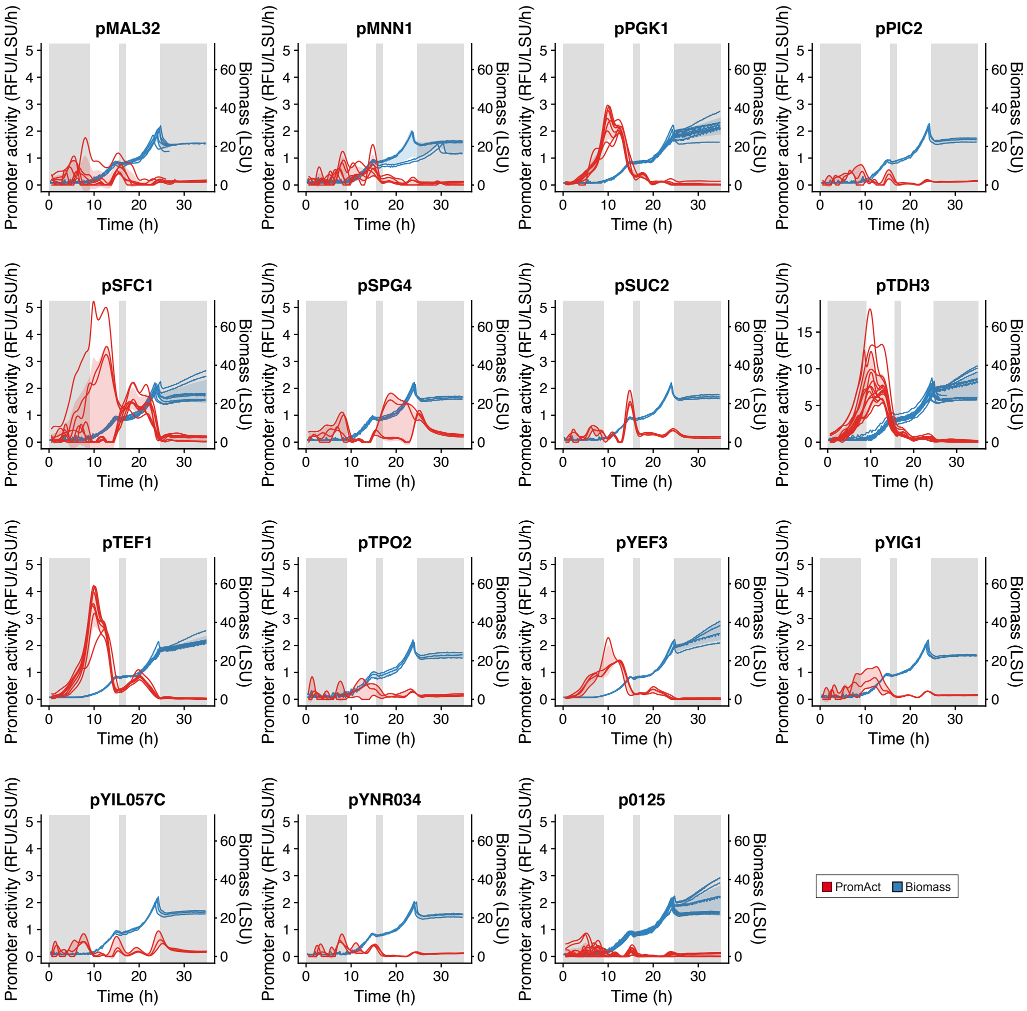

Figure S1. Activity of all promoters in batch culture. Number of replicates for each promoter detailed in Table S2.

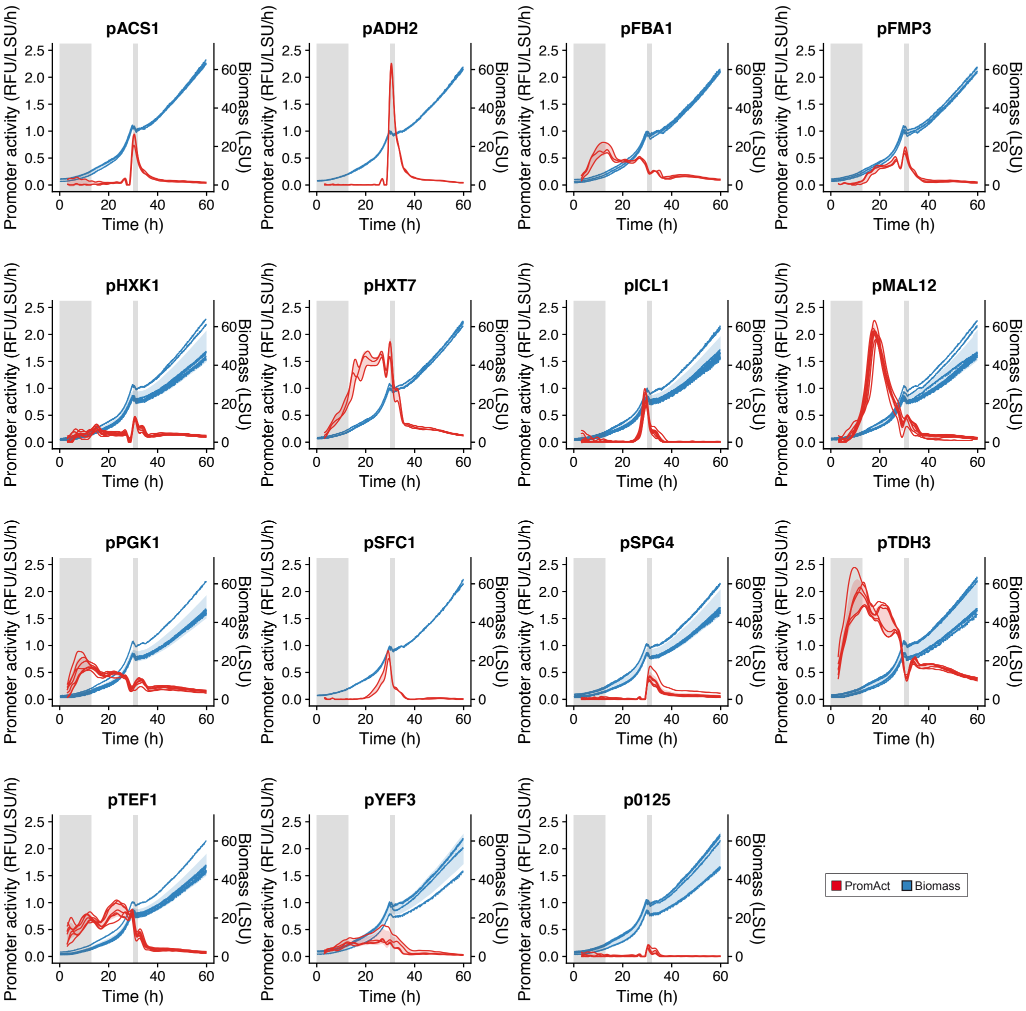

Figure S2. Activity of all promoters in fed-batch culture. Number of replicates for each promoter detailed in Table S3.
